# Molecular and Mechanistic Divergence of Seizures in Pediatric Acute Lymphoblastic Leukemia: CNS Infiltration Versus Chemotherapy-Induced Neurotoxicity

**DOI:** 10.3390/ijms27073307

**Published:** 2026-04-06

**Authors:** Jin Joo, Woo Sub Yang, Hyun Jung Koh

**Affiliations:** 1 Department of Anesthesiology and pain Medicine, Seoul St. Mary’s Hospital, College of Medicine, The Catholic University of Korea, Seoul 06591, Republic of Korea; jiyo1004@catholic.ac.kr; 2 Department of Genetics, Yale Stem Cell Center, Yale School of Medicine, New Haven, CT 06520, USA; woosubyang@gmail.com

**Keywords:** pediatric acute lymphoblastic leukemia (ALL), blood–brain barrier, chemotherapy-induced neurotoxicity, CNS leukemia, epilepsy, excitotoxicity, molecular biomarkers, leukemic infiltration

## Abstract

Neurological complications, particularly seizures, represent a significant and often under-recognized clinical challenge in pediatric hematologic malignancies. Distinguishing CNS leukemia-associated epilepsy from chemotherapy-induced neurotoxicity is critical for optimizing therapy but remains difficult due to overlapping clinical presentations. This review highlights the distinct molecular mechanisms underlying these two entities. CNS leukemia-associated seizures are primarily driven by blood–brain barrier (BBB) disruption following leukemic infiltration, which triggers a neuroinflammatory cascade involving pro-inflammatory cytokines such as IL-6 and TNF-α, and impairs glutamate homeostasis. In contrast, chemotherapy-induced seizures, particularly those associated with high-dose methotrexate, arise from disrupted folate metabolism, intracellular oxidative stress, and subsequent N-methyl-D-aspartate (NMDA) receptor-mediated excitotoxicity. We provide a comparative analysis of these pathways, integrating current evidence on pharmacogenomic susceptibility—including polymorphisms in methylenetetrahydrofolate reductase (MTHFR) and drug transporter genes—as well as epigenetic factors. By synthesizing these molecular insights, we propose a mechanistic framework for precise clinical differentiation, which may inform biomarker-driven diagnostic approaches and targeted neuroprotective strategies in this vulnerable population.

## 1. Introduction

With the intensification of risk-stratified therapeutic protocols, survival rates in pediatric leukemia—particularly acute lymphoblastic leukemia (ALL) and acute myeloid leukemia (AML)—now approach 90%. While this represents a major therapeutic milestone, it has concurrently shifted clinical priorities toward survivorship quality. Neurological complications have emerged as a significant determinant of long-term outcome, and among them, acute seizures represent one of the most alarming manifestations of central nervous system (CNS) dysfunction [1,2,3,4].

A critical challenge in pediatric oncology is the etiological ambiguity of seizures arising during leukemia treatment. An ictal event may originate from two diametrically opposed mechanisms: disease-driven CNS involvement, characterized by leukemic blast infiltration of the neurovascular niche, or treatment-driven neurotoxicity induced by intensive chemotherapy such as methotrexate or cytarabine. Although clinically overlapping, these entities arise from distinct molecular landscapes and require fundamentally different therapeutic strategies. Misclassification may lead to inappropriate treatment escalation or delayed recognition of CNS relapse, with significant consequences for both survival and neurological outcome. Conventional diagnostics—including neuroimaging, cerebrospinal fluid cytology, and electroencephalography—lack sufficient molecular resolution to reliably distinguish between these processes, underscoring the need for a mechanistically grounded approach [5,6,7].

Seizure generation ultimately reflects a lowering of the neuronal excitation threshold driven by disruptions in synaptic transmission, ionic homeostasis, neuroinflammatory signaling, and blood–brain barrier (BBB) integrity [4,6,8].

In leukemic CNS involvement, malignant blasts actively remodel the neuroimmune microenvironment. Secretion of pro-inflammatory cytokines such as interleukin-6 (IL-6) and tumor necrosis factor-α (TNF-α) activates microglia through NF-κB-dependent pathways, amplifying local inflammatory cascades. This inflammatory milieu disrupts astrocytic glutamate regulation, including the downregulation of excitatory amino acid transporter 2 (EAAT2), leading to extracellular glutamate accumulation and excitotoxicity. Concurrently, cytokine-mediated alterations in endothelial tight junction proteins such as claudin-5 and occludin compromise BBB integrity, facilitating further immune cell trafficking and enhancing cortical hyperexcitability. In this context, ictogenesis is closely linked to inflammatory neurovascular remodeling driven by malignant infiltration [7,8,9,10,11].

In contrast, chemotherapy-induced neurotoxicity arises in the absence of leukemic invasion and instead reflects sterile metabolic and oxidative injury. Methotrexate-associated elevations in homocysteine may potentiate N-methyl-D-aspartate (NMDA) receptor-mediated excitotoxic signaling, while cytotoxic agents promote reactive oxygen species (ROS) accumulation and mitochondrial dysfunction. Impaired ATP production destabilizes Na^+^/K^+^-ATPase activity, lowering seizure thresholds through membrane depolarization. Endothelial apoptosis and microvascular stress further weaken BBB integrity, but through oxidative and metabolic pathways rather than cytokine-dominant immune activation. Although both processes converge on excitatory–inhibitory imbalance, their upstream drivers—immune-inflammatory remodeling versus metabolic-oxidative stress—remain fundamentally divergent [9,12,13,14,15].

Despite growing recognition of seizure complications in pediatric leukemia, the current literature remains largely descriptive and lacks a systematic comparison of these distinct molecular landscapes. A unifying framework that integrates inflammatory signaling, excitotoxic mechanisms, mitochondrial vulnerability, and BBB dysfunction has yet to be established. In the era of precision oncology, where systemic therapies are increasingly biomarker-driven, a comparable molecular approach to neurological complications is urgently needed [5,7,10,11,14].

This review therefore proposes a mechanism-based differential framework to distinguish CNS leukemia-associated seizures from chemotherapy-induced neurotoxicity at the molecular level. By delineating convergent and divergent pathways of ictogenesis—including cytokine signaling, glutamatergic dysregulation, ionic dyshomeostasis, mitochondrial injury, and endothelial stress—we aim to provide a biologically informed foundation for precision diagnosis and targeted neuroprotective strategies. Clarifying the molecular divergence between these two diametrically opposed processes is essential for optimizing therapeutic decision-making and safeguarding long-term neurological outcomes in children with leukemia.

## 2. CNS Leukemia-Associated Epilepsy: Pathophysiological Mechanisms

### 2.1. MMP-2/9-Mediated Tight Junction Disruption and Blood–Brain Barrier Breakdown

**Figure 1:**
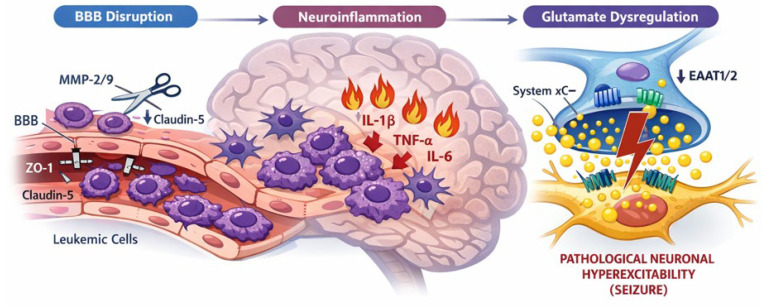
Pathophysiological Cascades Leading to CNS Leukemia-Associated Epilepsy. **Left:** MMP-mediated blood–brain barrier (BBB) breakdown. Leukemic blasts in the blood vessel secrete matrix metalloproteinases (MMP-2 and MMP-9), which proteolytically degrade key tight junction (TJ) proteins (ZO-1, Claudin-5), resulting in BBB breakdown and facilitating leukemic infiltration into the brain parenchyma. **Center:** Cytokine-driven neuroinflammation. Infiltrating leukemic cells and activated glial cells release pro-inflammatory cytokines (IL-1β, TNF-α, IL-6), which act on neurons and astrocytes to promote a pro-epileptogenic environment. **Right:** Glutamate cycle disruption and excitotoxicity. Leukemic cells increase extracellular glutamate via the cystine–glutamate antiporter (System xC^−^). Concurrently, neuroinflammation and metabolic stress downregulate astrocytic excitatory amino acid transporters (EAAT1/2), impairing glutamate clearance. The resulting pathological accumulation of extracellular glutamate leads to the overactivation of neuronal AMPA and NMDA receptors (with Src-dependent NR2B phosphorylation), along with the suppression of GABAergic currents. **Bottom:** These convergent processes culminate in sustained neuronal hyperexcitability and seizure generation.

Leukemic cell entry into the central nervous system is initiated by proteolytic remodeling of the blood–brain barrier (BBB). Leukemic blasts secrete matrix metalloproteinases (MMP-2 and MMP-9), which degrade key endothelial tight junction (TJ) proteins, including zonula occludens-1 (ZO-1), claudin-5, and occludin. In brain microvascular endothelial cells (BMVECs), this enzymatic cleavage disrupts junctional continuity and increases paracellular permeability [16,17]. A schematic overview of these pathophysiological cascades is presented in Figure 1.

In vitro BBB models demonstrate reduced TJ protein expression and increased permeability following exposure to leukemic cells. Consistently, in vivo leukemia mouse models show decreased ZO-1, claudin-5, and occludin levels accompanied by structural BBB opening. Importantly, RNA interference targeting MMP-2/9 or pharmacologic inhibition with the broad-spectrum MMP inhibitor GM6001 attenuates TJ degradation, restores barrier integrity, and reduces CNS leukemic burden [3,16].

These findings collectively position MMP-2/9-dependent tight junction proteolysis as a critical molecular gateway for CNS leukemic infiltration and the initiating event in leukemia-associated epileptogenic cascades [16,18,19].

### 2.2. Cytokine-Driven Neuroinflammatory Signaling and Synaptic Hyperexcitability

CNS leukemia-associated neuroinflammation is characterized by elevated IL-1β, TNF-α, and IL-6 in perivascular and parenchymal compartments, driven by leukemic cells and activated glia [20,21,22]. IL-1β rapidly enhances NMDA receptor function via Src-dependent NR2B phosphorylation and simultaneously depresses GABAergic currents, resulting in an acute shift toward neuronal hyperexcitability [22,23]. TNF-α promotes synaptic insertion of Ca^2+^-permeable AMPA receptors while internalizing GABA-A receptors, a form of pathological synaptic scaling that lowers seizure threshold [20,21,24,25]. Sustained IL-6 signaling disrupts hippocampal LTP, reduces neurogenesis, and promotes gliosis, thereby creating a permissive substrate for epileptogenesis rather than acting as a purely acute neuromodulator [20,24,26]. Even transient surges of IL-1β and TNF-α in seizure-prone regions can trigger long-lasting synaptic and structural remodeling, consolidating a chronically hyperexcitable network [20,22,27,28].

### 2.3. Astrocyte-Neuron Glutamate Cycle Disruption and Metabolic Excitotoxicity

CNS leukemia-associated epilepsy arises from disruption of the astrocyte–neuron glutamate–glutamine cycle, primarily through impaired excitatory amino acid transporters (EAATs). Astrocytic EAAT1/2 normally clear ~90% of synaptic glutamate, fueling glutamine synthesis for neuronal GABA/glutamate replenishment; their dysfunction elevates extracellular glutamate, reduces GABA, and lowers seizure threshold. In CNS leukemia, leukemic infiltration induces BBB breakdown, inflammation (IL-1β/TNF-α), and metabolic stress, downregulating astrocytic EAAT1/2 while promoting glial glutamate release, creating a pro-epileptogenic perivascular milieu [29,30,31]. This excitotoxicity may be further exacerbated by leukemic blasts’ metabolic dependence on glutaminolysis [32,33]. In this context, leukemic blasts may utilize the cystine–glutamate antiporter (system xC^−^) for redox balance [34,35]. Although direct evidence in CNS leukemia is limited, this mechanism is well-characterized in gliomas, where xCT overexpression increases extracellular glutamate, activates neuronal AMPA/NMDA receptors, and triggers epileptiform activity [36,37]. CSF glutamine elevation in pediatric CNS leukemia further supports amino acid dysregulation. Energy deficits from microvascular GLUT1 dysfunction impair Na^+^/K^+^-ATPase-driven EAAT uptake, amplifying excitotoxicity [30,38]. This leukemic glutamate microenvironment mechanistically distinguishes CNS involvement seizures (focal, chronic EAAT/system xC^−^ driven) from chemotherapy neurotoxicity (diffuse metabolic/white matter injury) [31,39].

## 3. Chemotherapy-Induced Epilepsy: Molecular Neurotoxicity

### 3.1. Methotrexate (MTX)-Induced Excitotoxicity

Methotrexate (MTX) competitively inhibits dihydrofolate reductase (DHFR), thereby disrupting folate-mediated one-carbon metabolism and leading to the systemic and intrathecal accumulation of homocysteine (HCY). Elevated HCY acts as a potent agonist at the glutamate-binding site of the NMDA receptor agonist, triggering excessive calcium influx and subsequent neuronal hyperexcitability that manifests clinically as seizures in pediatric leukemia patients [40,41]. This excitotoxic cascade manifests as seizures in pediatric leukemia patients, representing a metabolic derangement that is distinct from the focal inflammatory processes associated with CNS leukemic infiltration [9,42,43,44,45,46].

### 3.2. Vincristine-Mediated Axonal Transport Failure and Intrathecal Neurotoxicity

Vincristine disrupts microtubule dynamics by binding to tubulin heterodimers, thereby impairing both anterograde and retrograde axonal transport systems essential for neurotransmitter trafficking and synaptic homeostasis [47,48,49,50]. This transport failure compromises vesicular delivery, mitochondrial distribution, and neurotrophic signaling, ultimately destabilizing neuronal network integrity [51]. Intrathecal administration further exacerbates these effects by enabling direct CNS exposure, precipitating rapid axonal degeneration and ventriculitis [47,52]. In pediatric acute lymphoblastic leukemia (ALL), such toxicity manifests as diffuse white matter injury rather than focal leukemic infiltration. Unlike leukemic cell-mediated localized disruption, vincristine-induced damage produces widespread conduction failure and autonomic or cranial nerve dysfunction [47,48]. Collectively, microtubule destabilization-driven transport collapse lowers the seizure threshold through synaptic dysfunction, metabolic stress, and impaired neuronal signaling, providing a mechanistic framework distinct from infiltration-associated epileptogenesis [47,49,53,54].

### 3.3. Mitochondrial Dysfunction and Oxidative Stress

Exposure to MTX and vincristine catalyzes the production of reactive oxygen species (ROS), which overwhelms endogenous antioxidant defenses and compromises mitochondrial membrane integrity (Δψₘ) [55,56]. This bioenergetic failure, characterized by impaired ATP production and pro-apoptotic signaling, underlies the long-term neurotoxic sequelae observed in pediatric patients [57,58]. These mitochondrial-driven mechanisms represent a unique pathophysiological profile of chemotherapy-induced epilepsy, contrasting with the primary glutamate-driven pathways of CNS leukemia [56]. These mitochondrial-driven mechanisms represent a unique pathophysiological profile of chemotherapy-induced epilepsy, contrasting with the primary glutamate-driven pathways of CNS leukemia. While preclinical data support NMDA antagonists [46], prospective clinical trials specifically evaluating antioxidant or seizure prevention strategies remain needed.

**Figure 2:**
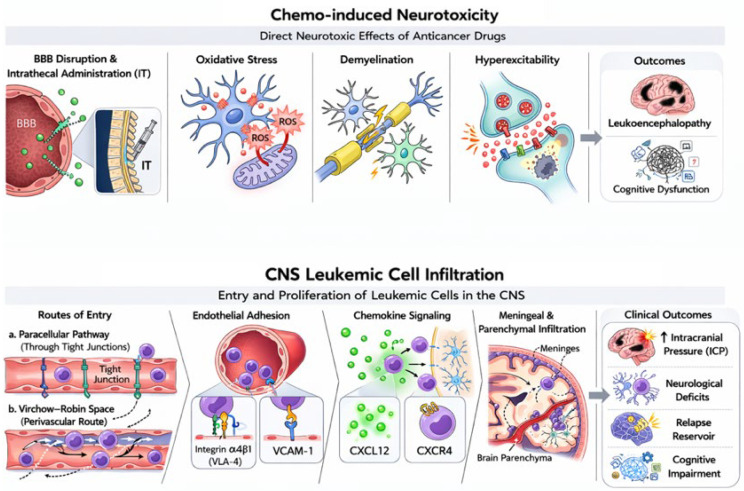
Dual Mechanistic Axes of CNS Injury in Pediatric Leukemia. **Upper panel:** Chemotherapy-induced neurotoxicity is characterized by metabolic and oxidative stress, including mitochondrial dysfunction, reactive oxygen species (ROS) accumulation, demyelination, and secondary neuroinflammation, ultimately leading to neuronal hyperexcitability and cognitive impairment. **Lower panel:** Leukemic blasts infiltrate the CNS through paracellular or perivascular pathways. Endothelial adhesion by integrin α4β1 (VLA-4)–VCAM-1 interactions promote migration and niche retention within the meningeal and parenchymal compartments. Together, these biologically distinct yet clinically convergent pathways contribute to neurological dysfunction, increased seizure susceptibility, and disease progression.

Figure 2 illustrates two biologically distinct yet clinically convergent mechanisms underlying central nervous system (CNS) complications in pediatric leukemia.

### 3.4. Additional Treatment-Related Neurotoxic Contributors

Beyond methotrexate and vincristine, other therapeutic modalities have been associated with seizure development in pediatric ALL. Cytarabine (Ara-C), particularly at high doses or via intrathecal administration, has been linked to cerebellar toxicity and acute encephalopathy, which may contribute to seizure occurrence [54]. L-asparaginase is associated with coagulopathy, increasing the risk of cerebral thrombosis or hemorrhage, both of which may precipitate seizures [59,60]. In addition, cranial irradiation has been implicated in delayed neurotoxicity, including white matter injury and chronic epileptogenic changes [61,62]. These mechanisms further underscore the heterogeneous etiologies of seizures in pediatric leukemia [6].

## 4. Differential Framework: Molecular Markers and Indicators

The distinction between leukemic infiltration and chemotherapy-induced neurotoxicity is often blurred in clinical practice due to overlapping neurological symptoms. However, their molecular underpinnings reveal distinct pathophysiological trajectories that necessitate precise biomarker-driven differentiation [47,48].

### 4.1. Comparative Analysis of Molecular Biomarkers

While both processes involve the disruption of the blood–brain barrier (BBB), the molecular drivers of infiltration are primarily “survival-centric” (for the blast cells), whereas toxicity markers are “injury-centric” (affecting neurons and glia) [55,56]. A comparative summary of these molecular and pathophysiological features is presented in Table 1.

This table summarizes the principal pathophysiologic orientations, blood–brain barrier interactions, metabolic alterations in cerebrospinal fluid (CSF), and representative structural biomarkers that may assist in differentiating leukemic CNS involvement from treatment-related neurotoxicity in pediatric ALL.

Importantly, this distinction is not merely conceptual but has direct diagnostic implications in clinical practice. Infiltration-associated processes are more likely to present with persistent cerebrospinal fluid abnormalities, progressive focal neurological deficits, and radiologic evidence of meningeal or parenchymal involvement. In contrast, chemotherapy-induced neurotoxicity often manifests as transient, diffuse, and potentially reversible white matter changes.

From a biomarker perspective, leukemic infiltration is typically associated with the upregulation of adhesion molecules (e.g., VLA-4, VCAM-1) and chemokine signaling axes (e.g., CXCL12–CXCR4), reflecting active leukemic trafficking and niche adaptation. Conversely, treatment-related neurotoxicity is more closely linked to structural injury markers such as neurofilament light chain (NfL), glial fibrillary acidic protein (GFAP), and S100B, which indicate axonal damage and astroglial injury. This mechanistic divergence provides a biologically grounded framework for differential diagnosis and supports the development of integrated molecular–clinical decision-making strategies.

### 4.2. Genetic Susceptibility: Polymorphisms Driving Inter-Patient Variability

Individual risk for neurotoxicity in pediatric ALL is heavily dictated by inherited genetic polymorphisms, particularly in folate metabolism and drug transport pathways [63,64].

#### 4.2.1. Methylenetetrahydrofolate Reductase (MTHFR)

The 677C > T and 1298A > C variants are the most studied. Reduced MTHFR activity may result in homocysteine accumulation, which has been associated with excitotoxic signaling through NMDA receptor overactivation, potentially contributing to white matter injury [57,63].

#### 4.2.2. Solute Carrier Organic Anion Transporter 1B1 (SLCO1B1)

This transporter facilitates the hepatic uptake of methotrexate (MTX). Variations such as rs4149056 (T > C) are associated with impaired MTX clearance, leading to higher systemic exposure and, consequently, an increased risk of acute leukoencephalopathy [5,64].

#### 4.2.3. ATP-Binding Cassette Subfamily B Member 1(ABCB1)

Encoding the P-glycoprotein (P-gp) efflux pump at the BBB, polymorphisms in ABCB1 (3435 C > T) can lead to altered P-glycoprotein-mediated efflux capacity across the BBB protection, allowing higher concentrations of chemotherapeutic agents to cross the BBB and accumulate in the CNS parenchyma [5,65].

### 4.3. Epigenetic Modifications: Developmental Sensitivity of the Pediatric Brain

The pediatric brain is not a static target; it is a landscape of rapid epigenetic remodeling. This makes it uniquely susceptible to chemotherapy compared to the adult brain [66,67].

#### 4.3.1. DNA Methylation Dynamics

Pediatric ALL survivors often exhibit “accelerated epigenetic aging”. Exposure to MTX can interfere with the methionine cycle, leading to global DNA hypomethylation in oligodendrocytes. This disruption prevents the normal developmental myelination process, manifesting as long-term cognitive deficits or “chemo-brain” [67,68].

#### 4.3.2. Histone Acetylation and Neural Plasticity

Chemotherapy-induced oxidative stress can alter histone acetyltransferase (HAT) activity. In the developing hippocampus, such modifications can silence genes responsible for synaptic plasticity (e.g., brain-derived neurotrophic factor, *BDNF*), explaining why executive function and memory are disproportionately affected in younger children [69].

#### 4.3.3. MicroRNA (miRNA) Dysregulation

Recent studies indicate that CSF-derived miRNAs (e.g., *miR-181*, *miR-146*) are modulated by treatment. These miRNAs regulate the inflammatory response of microglia; their epigenetic silencing can lead to chronic, low-grade neuroinflammation even after the cessation of chemotherapy [66].

These distinctions are not only mechanistically relevant but also have direct implications for clinical decision-making, particularly in guiding etiology-specific seizure management.

## 5. Clinical Implications and Emerging Strategies

Accumulating molecular evidence has improved our understanding of CNS complications in pediatric ALL. Translating these findings into clinical practice involves two parallel priorities: reducing treatment-related neurotoxicity and improving the early detection of CNS leukemic involvement. The following approaches reflect strategies discussed in the literature rather than established standards of care [70,71].

### 5.1. Targeted Prevention: Mechanism-Informed Neuroprotection

**Figure 3:**
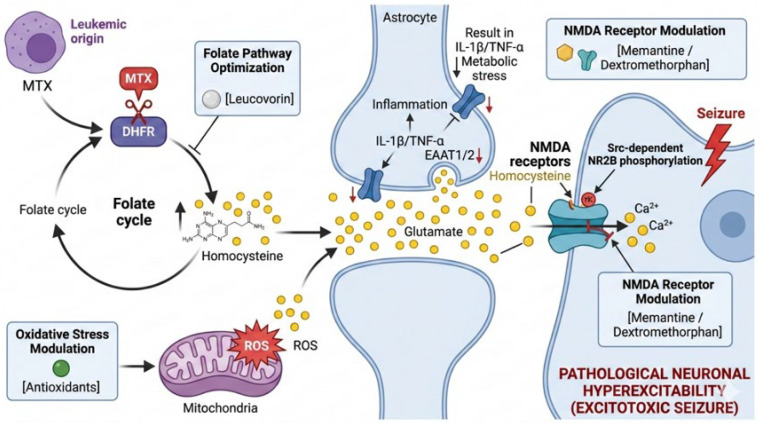
Mechanism-Informed Neuroprotection Strategies Targeting MTX-Induced Excitotoxicity. **Left & Center:** Metabolic dysregulation. Methotrexate (MTX) inhibits dihydrofolate reductase, disrupting the folate cycle and leading to homocysteine accumulation. MTX-induced mitochondrial dysfunction and ATP depletion impair Na+/K+-ATPase activity and astrocytic glutamate clearance via EAAT1/2. **Right:** Excitotoxic cascade. Excessive homocysteine acts as an NMDA receptor agonist, leading to excessive receptor activation (including Src-dependent NR2B phosphorylation) and calcium influx. This process is further amplified by extracellular glutamate accumulation, resulting in neuronal injury and death. Pharmacologic strategies include (1) Leucovorin rescue to restore folate metabolism, (2) Antioxidants approaches to mitigate oxidative stress, and (3) NMDA receptor antagonists (e.g., Memantine, Dextromethorphan) to attenuate the excitotoxic calcium influx (ROS, reactive oxygen species). Arrows indicate the direction of molecular and cellular processes. Yellow dots represent extracellular glutamate. Red lightning symbols denote pathological neuronal hyperexcitability and seizure activity. Blue structures indicate astrocytes and neuronal components involved in glutamate signaling.

Insights into MTX-associated metabolic and inflammatory pathways have prompted investigation into targeted neuroprotective strategies [70]. A schematic overview of these interconnected mechanisms—including folate-cycle disruption, oxidative stress, glutamate accumulation, and NMDA receptor–mediated excitotoxicity—is presented in Figure 3.

#### 5.1.1. NMDA Receptor Modulation

MTX exposure has been associated with homocysteine accumulation secondary to folate-cycle disruption. Elevated homocysteine levels have been linked to NMDA receptor-mediated excitotoxic signaling in experimental and clinical studies. Based on this mechanism, NMDA receptor antagonists such as memantine and dextromethorphan have been explored as potential adjunctive neuroprotective agents. However, clinical evidence in pediatric ALL remains limited, and further controlled studies are required before routine application can be considered [5,63].

#### 5.1.2. Folate Pathway Optimization

Leucovorin rescue is routinely used to reduce systemic MTX toxicity. Several studies have examined whether timing and dosing adjustments may influence CNS toxicity profiles. While optimization strategies have been proposed, maintaining the balance between neuroprotection and preservation of anti-leukemic efficacy remains a critical consideration, and consensus guidelines for CNS-specific modulation are not yet established [70,71].

#### 5.1.3. Oxidative Stress Modulation

Oxidative stress and mitochondrial dysfunction have been implicated in oligodendrocyte injury and white matter changes following CNS-directed chemotherapy. Antioxidant-based interventions have shown neuroprotective signals in preclinical models; however, consistent clinical data demonstrating long-term neurocognitive benefit in pediatric populations are currently insufficient [55,56].

### 5.2. Advanced Monitoring Beyond Conventional CSF Cytology

Cerebrospinal fluid cytology remains a central diagnostic tool for CNS leukemia but may lack sensitivity for early or low-level disease. Emerging molecular techniques aim to complement, rather than replace, conventional methods [72].

#### 5.2.1. CSF Liquid Biopsy Approaches

Detection of cell-free DNA (cfDNA), along with high-sensitivity flow cytometry for minimal residual disease (MRD) assessment in CSF, has demonstrated improved detection capability in several studies. These approaches may identify molecular CNS involvement prior to overt cytological relapse. Standardization of assay platforms to ensure inter-laboratory reproducibility and prospective validation are ongoing [72].

#### 5.2.2. Multiparametric Biomarker Panels

**Figure 4:**
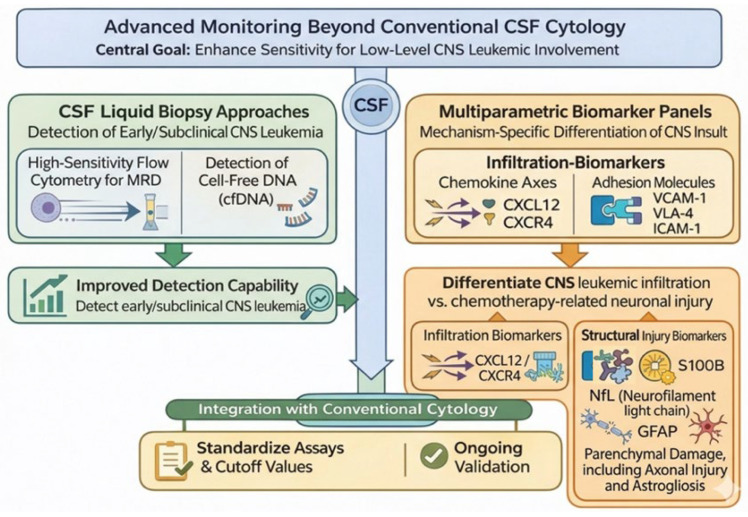
Advanced Diagnostic Framework for CNS Leukemia. **Left:** High-sensitivity molecular approaches, including cerebrospinal fluid (CSF) cell-free DNA (cfDNA) analysis and flow cytometry-based minimal residual disease (MRD) assessment, enable detection of subclinical CNS involvement beyond conventional cytology. **Right:** Integration of two distinct classes of molecular markers to differentiate etiologies of CNS injury. Infiltration-associated markers: Chemokine axes (e.g., CXCL12, CXCR4) and adhesion molecules (e.g., ICAM-1, VLA-4) reflect active leukemic cell trafficking and parenchymal infiltration. Structural injury biomarkers: Neurofilament light chain (NfL), GFAP, and S100B indicate parenchymal damage including axonal injury and astrogliosis. **Bottom:** This integrative framework supports clinical decision-making by distinguishing active leukemic infiltration from chemotherapy-induced neurotoxicity.

Integrating infiltration-associated markers (e.g., chemokine axes and adhesion molecules) with structural injury biomarkers such as neurofilament light chain (NfL), GFAP, and S100B has been proposed as a strategy to distinguish leukemic infiltration from chemotherapy-related neuronal injury. Although promising, defined cutoff values and longitudinal pediatric validation remain necessary for clinical implementation [55,73]. Figure 4 illustrates the transition from conventional CSF cytology to high-sensitivity molecular monitoring.

### 5.3. Current Limitations and Unresolved Challenges

**Figure 5:**
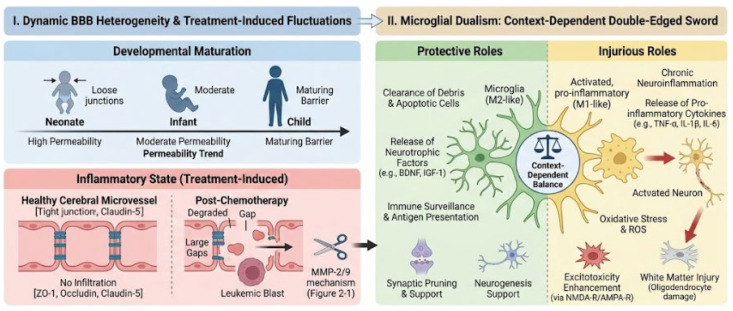
Unresolved Challenges in CNS-Directed Therapy. **Left:** Blood–brain barrier (BBB) permeability is a dynamic a dynamic parameter influenced by developmental stage and treatment-related factors. Chemotherapy can induce transient BBB disruption through proteolytic degradation mechanisms (e.g., MMP-2/9 mechanism), which currently lack real-time, non-invasive assessment tools. **Right:** Microglia activation exhibits a complex, dual function in CNS pathology. Protective (M2-like) responses support debris clearance and neurotrophic signaling, whereas injurious role (M1-like) activation promotes chronic neuroinflammation, oxidative stress, and excitotoxicity. Bridging molecular biomarkers with long-term neurocognitive outcomes requires integrated, longitudinal pediatric profiling frameworks.

Despite significant strides, several unresolved challenges remain in the management of CNS-directed therapy in pediatric ALL [71]. A conceptual overview of these challenges—including dynamic BBB heterogeneity across developmental stages and the context-dependent dual roles of microglial activation—is presented in Figure 5.

#### 5.3.1. Blood–Brain Barrier (BBB) Heterogeneity

BBB integrity varies across developmental stages and inflammatory states. Currently available clinical tools do not allow for the real-time, non-invasive assessment of dynamic BBB permeability. Genetic susceptibility markers (e.g., drug transporter polymorphisms) provide partial risk stratification but do not capture treatment-induced fluctuations [71].

#### 5.3.2. Persistent Epigenetic Alterations

Chemotherapy exposure during critical neurodevelopmental periods has been associated with lasting epigenetic modifications, including alterations in DNA methylation profiles. The long-term functional consequences and potential reversibility of these changes require further longitudinal investigation [66,67].

#### 5.3.3. Context-Dependent Neuroinflammation

Microglial activation has been reported to exert both protective and injurious effects depending on disease context and timing. Therapeutic modulation of neuroinflammation therefore requires careful evaluation to avoid unintended interference with CNS immune surveillance [71,72].

#### 5.3.4. Bridging Molecular Markers and Neurocognitive Outcomes

Although molecular and genetic biomarkers have been associated with neurotoxicity risk, direct correlations with standardized long-term neurocognitive outcomes remain incompletely defined. Development of integrated, longitudinal pediatric profiling frameworks incorporating molecular, imaging, and cognitive data represents an important future direction [71,74].

### 5.4. Emerging Immunotherapies and Neurotoxicity: CAR-T and CAR-NK

Recent advances in immunotherapy, particularly chimeric antigen receptor T-cell (CAR-T) therapy and emerging CAR-NK approaches, have significantly improved outcomes in relapsed or refractory hematologic malignancies, including pediatric ALL. However, these therapies are frequently associated with neurotoxicity, most notably immune effector cell-associated neurotoxicity syndrome (ICANS), which may manifest as seizures, encephalopathy, or focal neurological deficits [75,76].

The pathophysiology of CAR-T-related neurotoxicity is distinct from both leukemic infiltration and chemotherapy-induced injury. It is primarily driven by systemic cytokine release, endothelial activation, and blood–brain barrier (BBB) disruption. Elevated levels of IL-1β, IL-6, and TNF-α contribute to increased BBB permeability and neuroinflammation, creating a pro-excitatory environment similar to cytokine-driven epileptogenesis observed in CNS leukemia [77,78]. However, unlike leukemic infiltration, CAR-T-related neurotoxicity is typically reversible and not associated with direct malignant invasion [78,79].

These observations highlight an additional, clinically relevant category of seizure etiology in pediatric leukemia and underscore the importance of integrating immunotherapy-related neurotoxicity into the differential diagnostic framework [80,81].

### 5.5. Clinical Management of Seizures: A Mechanism-Based Approach

Management strategies for seizures in pediatric leukemia should be tailored according to the underlying etiology.

In cases of CNS leukemic infiltration, seizure management should prioritize rapid control of intracranial disease activity. This includes systemic or intrathecal chemotherapy intensification, corticosteroid administration to reduce inflammatory burden, and standard antiseizure medications for symptomatic control [6]. Early recognition is critical, as seizures in this context may reflect disease progression or relapse.

In contrast, chemotherapy-induced seizures are primarily driven by metabolic and excitotoxic mechanisms. Immediate management includes the correction of metabolic derangements, optimization of supportive care, and temporary modification or delay of neurotoxic agents. NMDA receptor antagonists and antioxidant strategies, as discussed above, may provide adjunctive neuroprotection, although clinical evidence remains limited [54].

Acute seizure episodes should be managed according to established pediatric neurological emergency protocols, including benzodiazepines as first-line therapy, followed by second-line agents such as levetiracetam or fosphenytoin where appropriate [6,54].

In this context, early etiological differentiation is critical, as inappropriate management may lead to either the undertreatment of CNS relapse or unnecessary modification of effective chemotherapy. For example, escalation of anti-leukemic therapy is essential in cases of true CNS infiltration, whereas similar intensification in chemotherapy-induced neurotoxicity may exacerbate neurological injury without clinical benefit.

Multidisciplinary collaboration among pediatric oncologists, neurologists, and critical care specialists is therefore essential for optimizing clinical outcomes, particularly in cases where overlapping mechanisms cannot be readily excluded.

The role of prophylactic anticonvulsant therapy remains controversial. Routine prophylaxis is not universally recommended; however, it may be considered in high-risk patients, such as those receiving intrathecal chemotherapy, experiencing recurrent neurotoxicity, or with prior CNS involvement. Decisions should be individualized based on clinical context and risk stratification [54]. Accurate etiological differentiation remains essential, as treatment priorities differ substantially across these conditions [6].

In addition, other etiologies such as CNS infection, intracranial hemorrhage, and treatment-related vascular complications should be considered, as these conditions may require distinct diagnostic and therapeutic approaches.

## 6. Conclusions and Future Directions

The management of CNS involvement in pediatric acute lymphoblastic leukemia (ALL) has entered a phase in which long-term survival must be considered alongside the preservation of neurocognitive function. Accumulating evidence indicates that leukemic infiltration and chemotherapy-induced neurotoxicity arise from biologically distinct molecular processes. Infiltration is characterized by active cellular migration and CNS niche adaptation, including chemokine-driven trafficking (e.g., CXCL12–CXCR4 signaling), whereas treatment-related neurotoxicity has been associated with metabolic perturbation, excitotoxic signaling, oxidative stress, and epigenetic dysregulation.

Despite advances in molecular characterization, important gaps remain. Future research will likely focus on three interconnected priorities. First, the validation of dynamic biomarkers, including the determination of the clinical utility of CSF-based liquid biopsies and structural injury markers such as neurofilament light chain (NfL) to differentiate subclinical relapse from iatrogenic injury. Second, pharmacogenomic integration by refining CNS-directed therapy stratification through the folate-pathway and drug transport polymorphisms, requiring standardized implementation frameworks. Third, long-term epigenetic consequences investigating whether early neuroprotective or epigenetically targeted interventions can mitigate persistent chemotherapy-associated neurocognitive “scars”.

Collectively, these efforts support a gradual shift toward a precision-oriented neuro-oncologic framework in pediatric ALL, in which molecular risk profiling, sensitive monitoring strategies, and neuroprotective considerations are integrated into CNS-directed treatment paradigms. Continued longitudinal and translational studies will be essential to align acute leukemia control with sustained neurodevelopmental health.

## Figures and Tables

**Table 1:** Key molecular distinctions between CNS leukemic infiltration and chemotherapy-induced neurotoxicity.

Category	CNS Leukemic Infiltration	Chemo-Induced Neurotoxicity (MTX/Ara-C)
Pathophysiologic Orientation [47]	Survival-centric (blast proliferation and CNS niche establishment	Injury-centric (neuronal and glial damage)
BBB Interaction [56]	VLA-4/VCAM-1 adhesion; CXCL12–CXCR4 transmigration	Endothelial activation; cytokine-mediated barrier disruption
CSF Metabolic Profile [57]	Lactate increase, Glucose decrease	Homocysteine increase, S-adenosylmethionine decrease
Structural Biomarkers [55]	CD19^+^/CD10^+^ leukemic blasts in CSF	NfL, GFAP, S100B increase
Extracellular Matrix [56]	MMP-2, MMP-9 (for basement membrane degradation)	GFAP as a marker of astrogliosis

BBB, Blood–Brain Barrier; VLA-4, Very Late Antigen-4; VCAM-1, Vascular Cell Adhesion Molecule-1; CXCL12, C-X-C Motif Chemokine Ligand 12; CXCR4, C-X-C Motif Chemokine Receptor 4; NfL, Neurofilament Light Chain; S100B, S100 Calcium-Binding Protein B; MMP, Matrix Metalloproteinase; GFAP, Glial Fibrillary Acidic Protein.

## Data Availability

No new data were created or analyzed in this study. Data sharing is not applicable.
